# CNBP restricts SARS-CoV2 by regulating IFN and disrupting RNA-protein condensates

**DOI:** 10.21203/rs.3.rs-1576788/v1

**Published:** 2022-05-02

**Authors:** Katherine Fitzgerald, Yongzhi Chen, Xuqiu Lei, Zhaozhao Jiang, Fiachra Humphries, Nicholas Mustone, Irene Ramos, Tinaye Mutetwa, Ana Fernandez-Sesma

**Affiliations:** University of Massachusetts Medical School; University of Massachusetts Medical School; University of Massachusetts Medical School; University of Massachusetts Medical School; UMass Chan Medical School; University of Massachusetts Medical School; Icahn School of Medicine at Mount Sinai; Icahn School of Medicine at Mount Sinai; Icahn School of Medicine at Mount Sinai

## Abstract

Severe acute respiratory syndrome coronavirus 2 (SARS-CoV-2) evades antiviral immunity through the expression of viral proteins that block detection, signaling, interferon (IFN) induction, and IFN-stimulated gene (ISG) expression^1, 2^. Weak induction of type I IFNs is associated with a hyperinflammatory response in patients that develop severe COVID-19^3, 4, 5^. Here we uncover a role for cellular nucleic acid-binding protein (CNBP) in restricting SARS-CoV-2. Typically, CNBP resides in the cytosol and, in response to RNA sensing pathways, undergoes phosphorylation, nuclear translocation, and IFNβ enhancer DNA binding to turn on IFNβ gene transcription. In SARS-CoV-2-infected cells CNBP coordinates IFNβ gene transcription. In addition, CNBP binds SARS-CoV-2 viral RNA directly. CNBP competes with the nucleocapsid (N) protein and prevents viral RNA and nucleocapsid protein from undergoing liquid-liquid phase separation (LLPS) forming condensates critical for viral replication. Consequently, cells and animals lacking CNBP have higher viral loads and CNBP-deficient mice succumb rapidly to infection. Altogether, these findings identify CNBP as a key antiviral factor for SARS-CoV-2, functioning both as a regulator of antiviral IFN gene expression and a cell intrinsic restriction factor that disrupts LLPS to limit viral replication and spread.

The ongoing COVID-19 pandemic has placed an enormous burden on public health and the global economy leading to >500 million infections and over 6 million deaths worldwide as of March 2022^6, 7, 8^. Severe acute respiratory syndrome coronavirus 2 (SARS-CoV-2), the causative agent of COVID19, is an enveloped, positive-sense single-stranded RNA virus^9, 10^. Infections with SARS-CoV-2 range from asymptomatic infection to severe and potentially fatal systemic inflammation, tissue damage, cytokine storm and acute respiratory distress syndrome. In infected epithelial cells, the virus is poorly detected by innate immune sensors due to antagonism of antiviral immunity^11, 12, 13, 14^. As a result, the induction of type I IFNs is weak and delayed^4, 5^ and in many individuals this is associated with more severe disease. A better understanding of the mechanisms that curb SARS-CoV-2 replication and host inflammatory responses are critical for understanding the variation in severity of COVID-19 and could provide new opportunities for prevention and treatment.

CNBP is a highly conserved DNA- and RNA-binding protein that is involved in gene transcription and translation^15, 16^. Previously, we identified CNBP as a key signaling molecule activated downstream of RNA-sensing pattern recognition receptors (PRRs) that control the transcription of type I IFNs to dsRNA and RNA viruses. CNBP is phosphorylated downstream of Toll-like receptors (TLRs) and RIG-I-like receptors (RLRs) by TGFbeta-activated protein kinase (TAK1), after which it moves to the nucleus where it binds the IFNβ enhancer together with IFN-regulatory factor 3 (IRF-3) to turn on the transcription of type I IFNs and antiviral responses^17^. Here we showed that, although SARS-CoV-2 infection leads to nuclear translocation of CNBP and reduced induction of type I IFNs, CNBP also binds SARS-CoV-2 viral RNA directly, interfering with a key step in the viral life cycle—blocking viral replication. In both cells and animals this leads to a reduction in viral loads with a profound influence on susceptibility to infection.

## Loss- and gain-of-function approaches indicate that CNBP inhibits SARS-CoV-2 replication *in vitro*

**Extended Data Figure 1:**
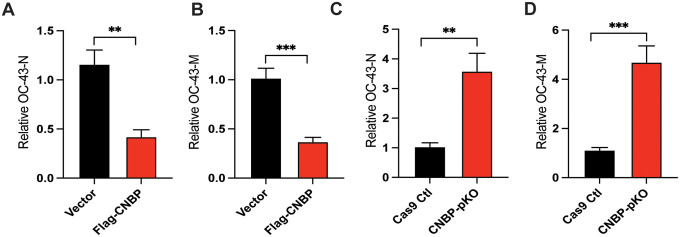
CNBP inhibits OC43 virus replication *in vitro* (A and B) Normalized OC43 RNA levels of OC43-N(A) and OC43-M(B) in hACE2-A549 cells transfected with Flag-CNBP plasmid and infected with OC43. (C and D) CNBP pKO and Cas9 Ctl A549 cells were infected with OC43 at an MOI of 0.01. qPCR analysis of viral RNA level of OC43-N(C) and OC43-M(D) at 24 h post-infection.

**Figure 1.**
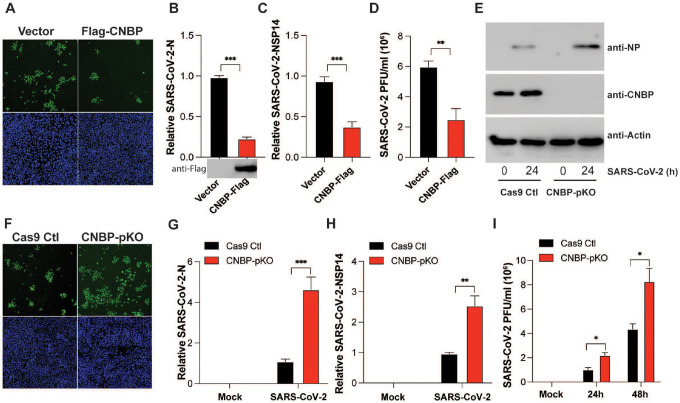
CNBP inhibits SARS-CoV-2 replication *in vitro* (A) hACE2-A549 cells were transfected with a Flag-CNBP expression plasmid or control, infected with SARS-CoV-2 for 24 hrs, and dsRNA was visualized by immunofluorescence with anti-J2 antibody (green). (B-D) Normalized SARS-CoV-2 RNA levels of NP (B) and NSP14 (C) as well as the SARS-CoV-2 titers (D) in hACE2-A549 cells transfected with Flag-CNBP plasmid and infected with SARS-CoV-2. (E-I) CNBP pKO and Cas9 Ctl A549 cells were infected with SARS-CoV-2 at an MOI of 0.01. At 24 h post-infection, western blotting with viral NP protein expression (E), immunofluorescence staining with anti-J2 antibody (F), qPCR analysis of vRNA levels of NP (G) and NSP14 (H) as well as the viral titers assessed by plaque assay (I) in the supernatants were determined.

Given our previous studies linking CNBP to antiviral immunity to other RNA viruses, we examined its role in controlling SARS-CoV-2 infection. A549-ACE2 expressing cells which are permissive to SARS-CoV-2 infection were transfected with CNBP or a vector control. We monitored the accumulation of double-stranded RNA using J2 antibody staining by immunofluorescence as a readout of virus infection and found the levels of J2 staining were reduced in cells overexpressing CNBP (Fig. 1A). Cells expressing CNBP also had reduced levels of viral N and NSP14 RNA and lower viral titers as measured by plaque assay relative to vector control cells (Fig. 1B–D). We also generated CNBP-deficient A549-ACE2 cells and after infection the levels of SARS-CoV-2 protein assessed using anti-NP antibodies was also higher in CNBP-deficient cells (Fig. 1E). Similarly, these cells had higher levels of J2 staining, N and NSP14 RNA levels, and had increased viral titers relative to wild-type (WT) cells (Fig. 1F–I). We observed similar effects with HCoV-OC43 infection, a related betacoronavirus (Extended Data Fig. 1A–D). Together, these data indicate that CNBP plays a role in limiting the replication of SARS-CoV-2 and related coronaviruses.

## CNBP limits SARS-CoV-2 infection via IFN-dependent and IFN-independent mechanisms

**Extended Data Figure 2:**
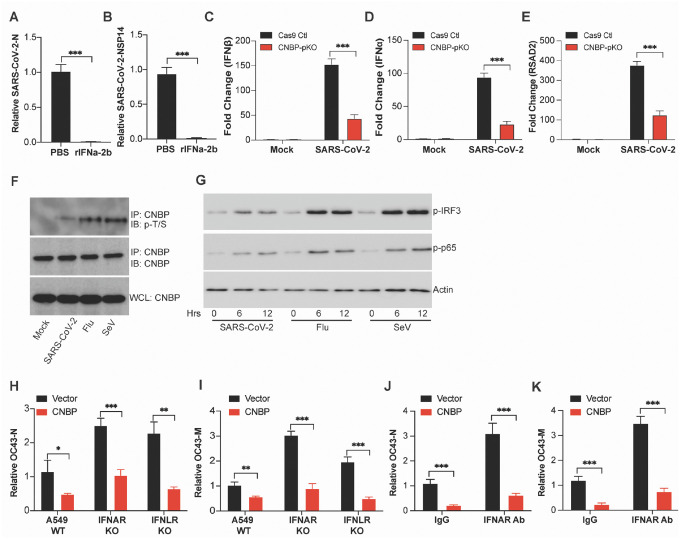
CNBP limits SARS-CoV2 infection via IFN-dependent and IFN-independent mechanisms. (A and B) Normalized SARS-CoV-2 RNA levels NP (A) and NSP14 (B) in A549-hACE2 cells pretreated with recombinant rIFNa-2b. (C-E) Normalized RNA levels of IFNβ (C), IFNα (D) and RSAD2 (E) in hACE2-A549 cells infected with SARS-CoV-2. (F) Endogenous CNBP protein was immunoprecipitated (IP) with anti-CNBP and immunoblotted (IB) with the anti–p-T/S for the phosphorylation of CNBP after treated with SARS-CoV-2, Flu or SeV. (G) Immunoblot analysis of p-IRF3 or p-p65 in whole-cell lysates of A549-hACE2 cells stimulated for various times with SARS-CoV-2, Flu or SeV as indicated. (H and I) Normalized OC43 RNA levels of N (H) and M (I) in IFNAR KO, IFNLR KO and Cas9 Ctl A549 cells transfected with Flag-CNBP. (J and K) Normalized OC43 RNA levels of N (J) and M (K) in A549 cells overexpressing Flag-CNBP treated with neutralizing antibody anti-IFNAR.

**Figure 2.**
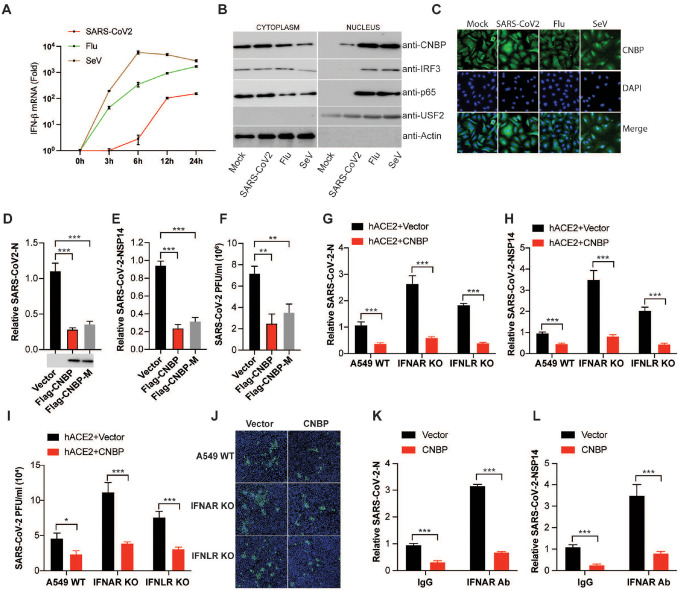
IFN-independent suppression of SARS-CoV-2 replication by CNBP (A) qPCR analysis of IFN-β mRNA induction by infection with SARS-CoV-2, Flu or SeV at different time points. (B) Immunoblot analysis of nuclear translocation of CNBP, IRF3 or p65 in A549 cells infected with SARS-CoV-2, Flu or SeV. (C) Localization of CNBP with or without SARS-CoV-2 infection as detected by immunofluorescence. (D-F) Normalized SARS-CoV-2 RNA levels of NP (D) and NSP14 (E), as well as the SARS-CoV-2 titers (F) in hACE2-A549 cells transfected with Flag-CNBP or Flag-CNBP mutant plasmid and infected with SARS-CoV-2. (G-J) IFNAR KO, IFNLR KO and Cas9 Ctl A549 cells co-transfected with a hACE2 plasmid with Flag-CNBP or Flag-CNBP-M were infected with SARS-CoV-2 at an MOI of 0.1. At 24 h post-infection, qPCR analysis of vRNA levels NP (G) and NSP14 (H), the viral titers (I) in the supernatants were determined by plaque assay and immunofluorescence staining with anti-J2 antibody (J). (K and L) qRT-PCR analysis of SARS-CoV-2 gRNA expression of NP (K) and NSP14 (L) in hACE2-A549 cells overexpressing CNBP treated with neutralizing antibody anti-IFNAR.

Infection of A549-ACE2 cells with SARS-CoV-2 leads to a delayed IFNβ response that is weak relative to that seen with either influenza or Sendai viruses (Fig. 2A). Treating SARS-CoV-2-infected cells with recombinant IFNα led to a marked decrease in viral RNA levels, indicating that SARS-CoV-2 is sensitive to type I IFN treatment (Extended Data Fig. 2A–B). The levels of IFNβ, IFNα and RSAD2 (Viperin) in SARS-CoV-2-infected A549-ACE2 cells were decreased in cells lacking CNBP, indicating that CNBP contributes to these responses (Extended Data Fig. 2C–E). Endogenous CNBP is predominantly localized in the cytoplasm at steady state and phosphorylated and translocated into the nucleus after influenza or SeV treatment (Fig. 2B and Extended Data Fig. 2F). In SARS-CoV-2-infected cells, however, the nuclear translocation and phosphorylation of CNBP was only weakly observed. Under these conditions, there was weak phosphorylation and translocation of IRF3 or p65, consistent with weak antiviral sensing in these cells (Extended Data Fig. 2G). Further, immunofluorescence microscopy showed that CNBP was retained in the cytosol of SARS-CoV-2-infected cells (Fig. 2C). Together, these results demonstrate that the IFN/ISG response in SARS-CoV-2-infected cells depends on CNBP.

Previous work from our lab and others demonstrated that CNBP is phosphorylated by TAK1 kinase which in turn controls its nuclear translocation^17, 18^. A phosphorylation defective T173/177A mutant is retained in the cytosol and fails to regulate the type I IFN response. We therefore tested if the mutant of CNBP could still restrict SARS-CoV-2 replication in transfected A549-ACE2 cells. To this end, we transfected the WT and CNBP mutant (CNBP-M) and monitored SARS-CoV-2 infection (Fig. 2D–F). The CNBP-M was just as effective as the WT in blocking infection, suggesting that CNBP still inhibits SARS-CoV-2 infection independent of its role as a signaling molecule controlling type I IFN gene expression. Consistent with this finding, overexpression of CNBP still blocked SARS-CoV-2 replication in IFN α/β receptor (IFNAR) KO A549 ACE2 cells (Fig. 2G–J). Similar results were obtained when A549-ACE2 cells lacking the IFNλ receptor (IFNLR) were used. Further, overexpression of CNBP blocked SARS-CoV-2 replication in cells treated with an anti-IFNAR antibody (Fig 2K–L). Similar results were obtained using the human coronavirus OC43 (HCoV-OC43) (Extended Data Fig. 2H–K). These results indicate that CNBP halts SARS-CoV-2 replication through induction of type I IFN but also through IFN-independent mechanisms.

## CNBP binds viral RNA competing with NP and leading to disruption of viral RNA-nucleocapsid protein condensates

**Extended Data Figure 3:**
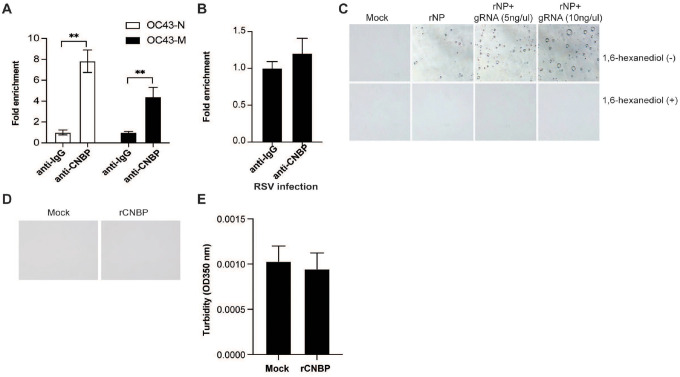
SARS-CoV-2 N undergoes LLPS (A and B) RIP assay with hACE2-A549 cell lysates prepared after 24 h of infection with OC-43 virus or respiratory syncytial virus (RSV) by using anti-CNBP or control immunoglobulin. Immunoprecipitated OC-43 virus RNA (A) or RSV RNA (B) was quantified by RT-qPCR. (C) Nucleoprotein LLPS in the presence of SARS-CoV2 genome RNA observed under bright field using a confocal microscope and were disrupted in the presence of 1,6-hexanediol. (D) 20 μM CNBP fails to undergo LLPS. (E) The turbidity of CNBP was measured by absorbance at 350 nm.

**Figure 3.**
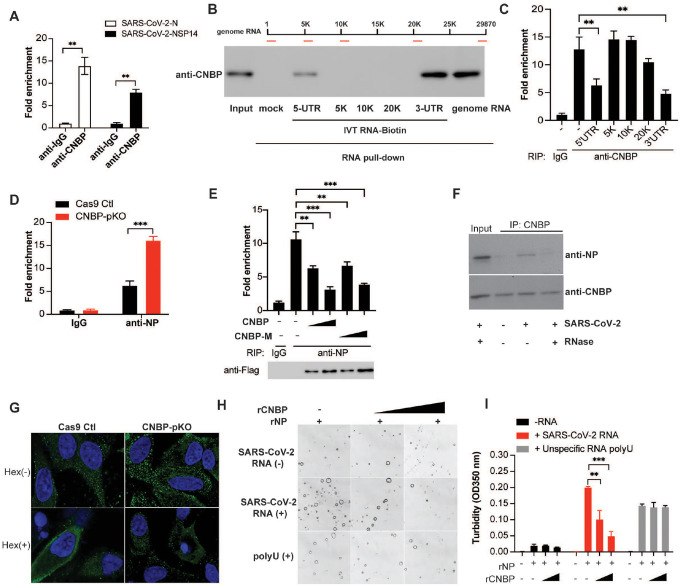
CNBP binds viral RNA competing with NP leading to disruption of viral RNA-nucleocapsid protein condensates (A) RIP assay with hACE2-A549 cell lysates prepared after 24 h of infection with SARS-CoV-2 by using anti-CNBP or control immunoglobulin. Immunoprecipitated SARS-CoV-2 positive-strand RNA was quantified by RT-qPCR. (B) RNA pull-down assay showing the binding activity of SARS-CoV-2 RNA genome or in vitro-transcribed (IVT) RNAs to CNBP. (C) RIP assay and RT-qPCR analysis of the binding activity of CNBP with SARS-CoV-2 genome RNA in the present of the indicated IVT RNAs. (D) RIP assay with A549 WT or CNBP pKO cell lysates prepared after 24h of infection with SARS-CoV-2 by using anti-NP. The immunoprecipitated SARS-CoV-2 positive-strand RNA was quantified by RT-qPCR. (E) CNBP pKO transfected with CNBP and CNBP-M, cell lysates were prepared after 24h of infection with SARS-CoV-2, the interaction of SARS-CoV-2 positive-strand RNA with NP was analyzed by RIP assay and RT–qPCR analysis as described in D. (F) Co-immunoprecipitation of CNBP and NP protein in SARS-CoV-2-infected cell lysates treated with or without RNase. (G) Increased NP puncta are formed in CNBP pKO cells compared with Cas9 Ctl hACE2-A549 cells infected with SARS-CoV-2 and disrupted by treating cells with 1,6-hexanediol. (H) NP protein LLPS were observed under bright field of a confocal microscope and could be disrupted by the addition of rCNBP. (I) The turbidity of each sample was measured by absorbance at 350 nm.

We next wanted to understand how CNBP curbs SARS-CoV-2 infection through IFN-independent mechanisms. Two independent groups reported an unbiased analysis of host proteins that bind to SARS-CoV-2 viral RNA. CNBP was the top SARS-CoV-2 genomic RNA-host binding protein identified in these studies^19, 20^. We therefore considered the possibility that CNBP bound viral RNA directly. We confirmed that CNBP directly binds SARS-CoV-2 viral RNA by performing RNA immunoprecipitation (RIP) followed by qPCR to quantify viral RNA levels (N and NSP14 RNAs). SARS-CoV-2 viral RNA was enriched in the CNBP pulldowns (Fig. 3A). CNBP could also bind RNA from HCoV-OC43 but not respiratory syncytial virus (Extended Data Fig. 3A and B). We next mapped the region(s) of SARS-CoV-2 genomic RNA that was bound by CNBP. We generated biotin-labeled RNAs corresponding to the 5′ UTR, 3′ UTR and three internal regions by in vitro transcription (IVT) and used these in pulldown experiments. CNBP was enriched in the streptavidin pulldowns using both the 5′ UTR and 3′ UTR RNA fragments but not by the RNA fragments corresponding to internal regions of the genomic RNA (Fig. 3B). We also performed the anti-CNBP RIP qPCR experiments in infected cells and showed that endogenous CNBP binding to SARS-CoV-2 genomic RNA was reduced by incubating these pulldown reactions with IVT RNAs corresponding to the 5′ UTR and 3′ UTR but not by IVT RNAs from other regions of the genomic RNA (Fig. 3C).

The SARS-CoV-2 nucleocapsid protein is an RNA-binding protein that plays a critical role in viral genome packaging and virion assembly. We speculated that CNBP might compete with the N protein for viral RNA. We confirmed viral RNA binding to the N protein by RIP-qPCR. Anti-NP pulldowns demonstrated that NP bound viral RNA in infected cells and NP binding to RNA was elevated in cells lacking CNBP (Fig. 3D). Further, overexpression of CNBP or the CNBP T173/177A mutant blocked the binding of the N protein to viral RNA in a dose-dependent manner (Fig. 3E). We could also detect N protein associated with CNBP during SARS-CoV-2 infection; however, the interaction between CNBP and SARS-CoV-2 N was sensitive to RNase digestion, suggesting that CNBP and SARS-CoV-2 N form a complex in the presence of viral RNA (Fig. 3F).

Recently, several independent groups have reported that NP can undergo liquid-liquid phase separation (LLPS) in the presence of viral genomic RNA, and the formation of these RNA-protein condensates increases the efficiency of viral RNA transcription and assembly of virions^21,22, 23, 24^. The 5′ UTR and 3′ UTR are important in the formation of these RNA-NP condensates^25,26^. We confirmed that NP forms condensates in the presence of increasing concentrations of viral RNA and the NP-RNA condensates were dissolved by 5% 1,6-hexanediol, an organic solvent known to disrupt a wide range of biomolecular condensates (Extended Data Fig. 3C). A549-ACE2 cells showed the formation of N protein puncta after SARS-CoV2 infection and the formation of these puncta was enhanced in CNBP-deficient cells (Fig 3G). These puncta could be disrupted by treating cells with 1,6-hexanediol. The high level of N protein puncta in CNBP-deficient cells prompted us to test whether CNBP modulates LLPS of NP in vitro. As expected, CNBP itself failed to undergo LLPS (Extended Data Fig.3D–E). NP in the presence of viral RNA formed droplets and recombinant CNBP inhibited the formation of these droplets—both the size and number of droplets decreased (Fig. 3H–I). The suppressive effect of CNBP was dose dependent in this assay as shown by quantifying the turbidity at 350 nm. Interestingly, the nonspecific polyU homopolymer RNA also induced LLPS of NP; however, these condensates were not impacted by CNBP (Fig. 3H–I). Collectively, these data demonstrate that SARS-CoV-2 NP undergoes RNA-induced LLPS and this process is disrupted by CNBP.

## CNBP inhibits SARS-CoV-2 infection *in vivo*

**Extended Data Figure 4:**
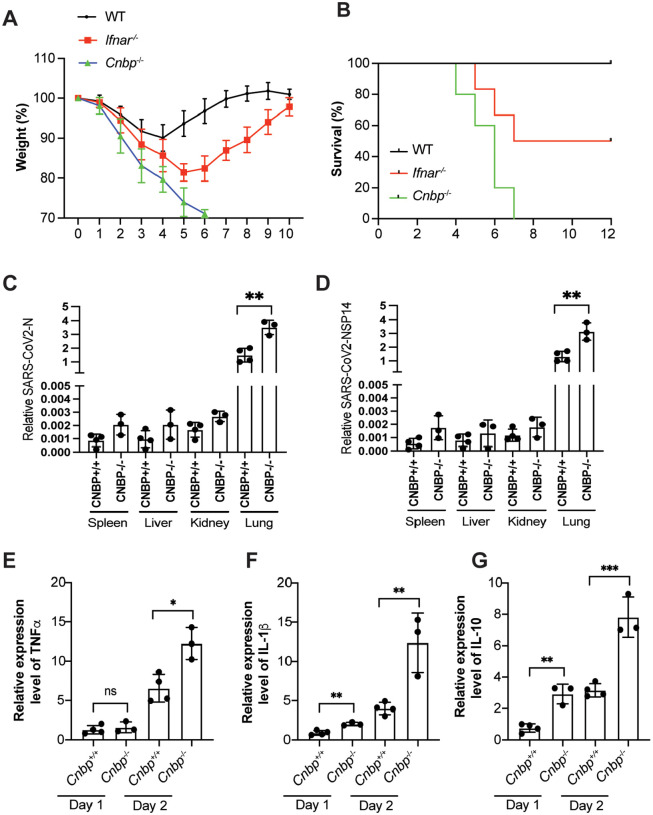
CNBP inhibits SARS-CoV2 infection in vivo (A and B) Weight loss (A) and survival (B) of Ifnar−/− and Cnbp−/− mice intranasally infected with SARS-CoV-2 MA10 strain (1*10e5 PFUs). (C and D) qRT-PCR analysis of SARS-CoV2 virus RNA levels NP(C) and NSP14 (D) in variant tissues. (E-G) Normalized mRNA levels of TNFα (E), IL1β (F) and IL-10 (G) from lung samples of mice infected with SARS-CoV-2 MA10 strain.

**Figure 4.**
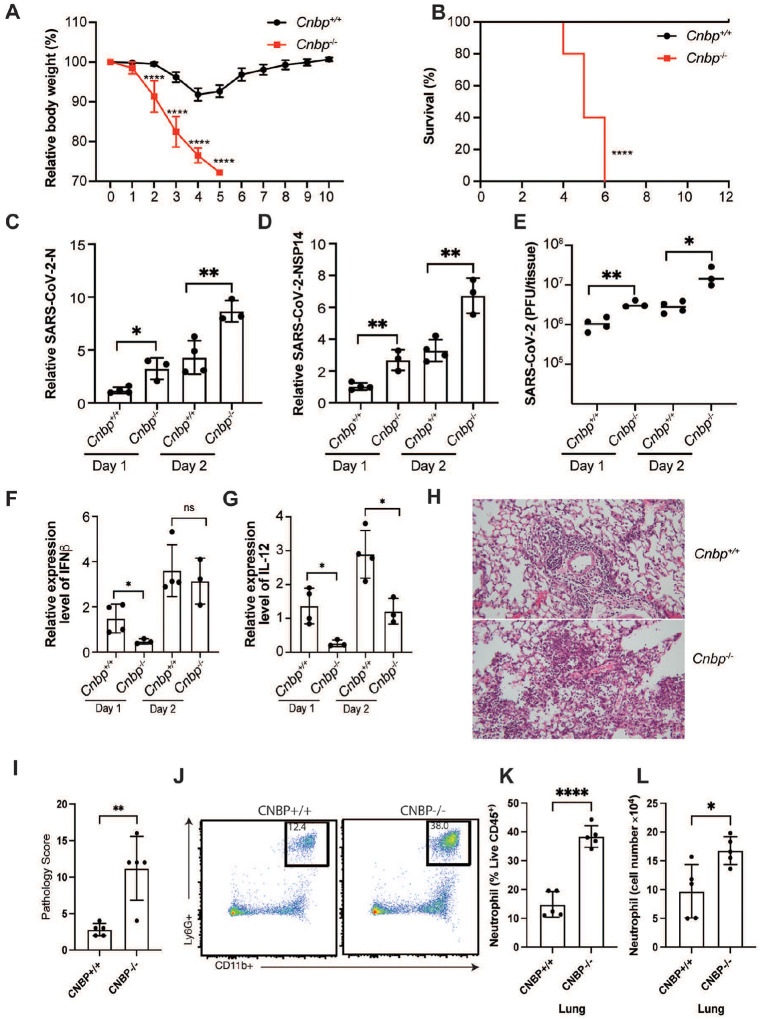
CNBP inhibits SARS-CoV-2 infection in vivo (A and B) Weight loss and survival of WT and *Cnbp*^−/−^ mice intranasally infected with SARS-CoV-2 MA10 strain (1*10e5 PFUs). (C-D) WT and *Cnbp*^−/−^ mice were infected intranasally with SARS-CoV-2 MA10 strain (1*10e5 PFUs) on day 1 and 2 post-infection (p.i.), the lungs were collected for qRT-PCR analysis of virus RNA levels NP(C) and NSP14 (D). (E) Viral lung titers of WT and *Cnbp*^−/−^ mice at 1 and 2 days p.i. (F and G) Normalized mRNA levels of IFN-β (F) and IL12b (G) from lung samples infected with SARS-CoV-2 MA10 strain. (H and I) Representative images (H) and pathology evaluation (I) of H&E-stained lung sections from WT and *Cnbp*^−/−^ mice at 4 days p.i. of SARS-CoV-2 MA10. (J-L) Flow plots (J), percentage (K) and cell number (L) of neutrophils in the lung from WT and *Cnbp*^−/−^ mice at 4 days p.i.

We next tested if CNBP was important in restricting SARS-CoV-2 *in vivo* by infecting CNBP-deficient mice and WT littermate controls. We used a mouse-adapted SARS-CoV-2 MA10 variant (ic2019-nCoV MA10) that efficiently infects C57BL/6 mice^27^. WT and CNBP-deficient mice were infected with MA10 (1×10^5^) and monitored for weight loss and survival over the course of 10 days. Wild-type animals exhibited transient weight loss (5–10%) after infection and recovered rapidly. In contrast, *Cnbp*^−/−^ mice lost weight rapidly and all succumbed to the infection within 6 days (Fig. 4A and B). The susceptibility of CNBP-deficient mice was more pronounced than that seen in *Ifnar* KO mice. While 100% of the *Cnbp* KO mice succumbed to SARS-CoV-2 infection, only ~50% of the *Ifnar* KO mice succumbed to the infection at this dose (Extended Data Fig. 4A–B). The more pronounced susceptibility of CNBP-deficient mice relative to IFNAR-deficient mice provide additional support for both IFN-dependent and IFN-independent functions of CNBP in restricting SARS-CoV-2. We also monitored RNA levels and viral titers in the lungs 1- or 2-days post infection (dpi) and found that the levels of viral RNA or viral titers were higher in *Cnbp*^−/−^ mice compared to the wild-type littermate controls (Fig. 4C–E). We also detected slightly higher viral RNA in the spleen, liver and kidney of CNBP-deficient mice than in WT mice, although the infection was still largely contained to the lung (Extended Data Fig. 4C–D). Consistently, we detected reduced IFN-β and interleukin-12 p40 (IL12p40) mRNAs in *Cnbp*^−/−^ mice at early time points (Fig. 4F and G); however, these KO mice had elevated TNF-α, IL-1β, and IL-10 mRNA, compared with WT mice (Extended Data Fig. 4E–G). Histopathological analysis was also performed on the lungs of mice infected with SARS-CoV-2 MA10. At 4 dpi, WT mice had evidence of alveolar septal thickening and mild inflammatory cell infiltration, whereas *Cnbp*^−/−^ mice showed severe alveolar septal thickening and infiltration of immune cells (Fig. 4H and I). Flow cytometry demonstrated that neutrophil recruitment to the lungs was also elevated in CNBP KO mice, while other immune cells showed no significant differences (Fig. 4J–L).

## Discussion

Patients with genetic mutations in antiviral genes that control production of type I IFNs suffer from life-threatening COVID-19 disease^4, 28^. Further, autoantibodies that neutralize type I IFNs have also been identified in patients and correlated with more severe COVID-19 disease^29^. Collectively, these observations highlight the important role innate antiviral responses play in curbing the replication of SARS-CoV-2. Here, we identify CNBP as a key host factor controlling SARS-CoV-2 infection. Consistent with its role in other RNA virus infections, CNBP coordinates signaling events that couple RNA sensing to type I IFN gene transcription. Cells lacking CNBP or receptors for type I or type III IFNs have elevated viral loads, and animals lacking IFNAR are more susceptible to virus infection than their wild-type counterparts.

Although SARS-CoV-2 is sensitive to exogenous type I IFN treatment, like many other viruses, SARS-CoV-2 deploys a range of countermeasures to subvert type I IFN responses to overcome innate antiviral defenses^30, 31^. SARS-CoV-2 is particularly adept at evading host innate immunity, and as a consequence very low levels of type I IFNs are detected in the lungs or blood of infected patients compared to that seen with other viruses^32, 33^. Indeed, our in vitro data also demonstrated that SARS-CoV-2 induces weak and delayed type I IFNs and ISGs in infected cells compared with other viruses. Consistently, there was weak nuclear translocation and phosphorylation of IRF3, p65 and CNBP, suggesting that CNBP is poorly activated in SARS-CoV-2-infected cells likely due to a failure of RNA sensors to appropriately recognize the virus and induce downstream signaling.

As a consequence of limited RNA sensing in SARS-CoV-2-infected cells, only a small amount of CNBP translocates to the nucleus to turn on type I IFNs. Most of the CNBP is retained in the cytosol where it could still inhibit SARS-CoV-2 replication. Indeed, our data suggests that CNBP acts in a cell intrinsic manner to restrict virus replication. The association of the N protein with viral genomic RNA leading to higher-order RNA-protein complexes is a key step in the replication of SARS-CoV-2, serving to concentrate RNA and proteins during virion assembly. CNBP targets this essential step by disrupting the phase separation that occurs with viral RNA and N proteins. Mechanistically, CNBP binds SARS-CoV-2 viral genomic RNA and precludes the N protein from forming condensates. CNBP binds the 5′ UTR and 3′ UTR and these regions are known to be important for the LLPS observed with NP-RNAs. Thus, the current findings demonstrate that CNBP disrupts the LLPS of the N protein and highlight the SARS-CoV-2 N protein LLPS as a promising therapeutic target during SARS-CoV-2 infection. Indeed, several small molecules have been reported to inhibit viral replication by targeting LLPS of viral N proteins^25, 34, 35, 36^. However, to our knowledge, CNBP is the first host factor that impacts viral replication through targeting viral-specific RNA sequences required for LLPS revealing a novel host directed antiviral strategy. Consistent with the impact of CNBP in controlling type I IFNs and its impact on RNA-NP condensates, we observed a marked susceptibility of CNBP-deficient mice to SARS-CoV-2 infection. The impact of CNBP-deficiency was greater than that seen in IFNAR-deficient mice, underscoring the dual function of CNBP.

Recent work has highlighted how the N protein RNA condensates contribute to viral transcription, replication, and immune evasion by targeting the mitochondrial antiviral-signaling protein (MAVS) as a mechanism to disrupt type I IFN signaling^37^. Further, SARS-CoV-2 N LLPS facilitates NF-κB hyper-activation and inflammation through regulation of TAK1 and IκB kinase (IKK)^38^. Our results also demonstrated that CNBP positively regulates type I IFN expression during RNA virus infection. Whether the disruption of the N protein LLPS by CNBP could restore innate antiviral immunity at the level of MAVS warrants further study.

A detailed understanding of the molecular mechanisms involved in restricting SARS-CoV-2 infection and how SARS-CoV-2 attempts to disrupt these mechanisms could reveal new therapeutic opportunities to boost antiviral mechanisms and clear SARS-CoV-2. Altogether, our findings underscore the importance of CNBP during SARS-CoV-2 infection highlighting the importance of this factor as a regulator of type I IFNs and antiviral responses and as a cell intrinsic restriction factor. The discovery of distinct functional outcomes of CNBP depending on its cellular location, provide important new insights that could be leveraged to improve the outcome of host interactions with this potentially deadly pathogen.

## Methods

### Biosafety

All study protocols were reviewed and approved by the Environmental Health and Safety and Institutional Review Board at the University of Massachusetts Chan Medical School prior to study initiation. All experiments with SARS-CoV-2 were performed in a biosafety level 3 laboratory by personnel equipped with powered air-purifying respirators.

### Viruses

Vero E6 cells were infected with the USA-WA1/2020 (NR-52281; BEI Resources) or the mouse-adapted MA10 variant of SARS-CoV-2 (in isolate USA-WA1/2020 backbone), Infectious Clone (ic2019-nCoV MA10) from ATCC. Supernatants were centrifuged at 450 *g* for 10 min and aliquoted and stored at −80°C. HCoV-OC43 was obtained from Dr. William M. McDougall (UMass Chan Medical School), RSV was obtained from Dr. Robert W. Finberg (UMass Chan Medical School), and SeV (Cantell strain) was purchased from Charles River Laboratories. Virus titer was determined by a TCID_50_ assay in Vero E6 cells. For the purification of genomic SARS-CoV-2 RNA (gRNA), the supernatant from Vero cells infected with SARS-CoV-2 was lysed in TRIzol LS, and viral RNA was extracted from the TRIzol using chloroform extraction.

### Cell culture

Human ACE2-A549 cells were a gift from Dr. Benjamin TenOever (NYU Langone Virology Institute), and Vero E6 cells or Hek293 cells cultured in Dulbecco’s modified Eagle’s medium supplemented with 10% (v/v) fetal bovine serum, 100 U/ml penicillin and 100 μg/ml streptomycin.

### CRISPR/Cas9 KO

Human ACE2-A549 cells or Hek293 cells were seeded on 6-well plates; after 16 h, plasmids expressing Cas9 and single-guide RNA (sgRNA) were cotransfected into cells. At 36 h after transfection, cells were selected for puromycin and blastomycin resistance for another 72–96 hours, then cells were passaged for 1–2 weeks prior to experimental use. Targeting of the desired gene was evaluated by western blot for loss of endogenous protein. sgRNA sequences are shown in Table S1. Generation of CRISPR IFNAR1 KO A549 cells has been previously described^39^. For the generation of IFNLR1 KO A549 cells, CRISPR-Cas9 ribonucleoprotein (RNP) complex (IDT) were transfected using the Nucleofector system (Lonza Bioscience). A predesigned Alt-R CRISPR-Cas9 gRNA targeting exon 3 (design ID: Hs.Cas9.IFNLR1.1.AA), the ATTO 550 Alt-R CRISPR-Cas9 tracrRNA, and the Alt-R S.p. HiFi Cas9 Nuclease were used to form RNPs in vitro

### Co-immunoprecipitation and Western Blot Analysis

Cell lysis and immunoblot analysis were performed as described previously^17^.

### In vitro phase separation assays

Phase separation of N protein (in 5 mM HEPES, pH 7.5, 100 mM NaCl) was induced by adding SARS-CoV-2 genomic RNA with increasing concentrations of CNBP protein. Samples were mixed and then immediately transferred onto microscope glass slides. Condensates were imaged within 10–20 min or as indicated in the experiment.

### Turbidity measurements

Turbidity was used to evaluate the phase separation of SARS-CoV-2 NP protein at different conditions determined using a NanoDrop spectrophotometer. Increasing concentrations of CNBP were added immediately before the experiments, followed by thoroughly pipetting and measurement of turbidity by absorbance at 350 nm. Average turbidity values were derived from measurements of three independent, freshly prepared samples.

### RNA Immunoprecipitation (RIP)-qPCR

Human ACE2-A549 cells were infected with SARS-CoV-2 (MOI=1) for 24 h, then the cells were fixed using 4% PFA for 15 min. Cell lysates were immunoprecipitated with IgG, anti-CNBP or anti-NP and protein G beads at cold room for overnight. The bead-bound immunoprecipitants were washed 3 times with lysis buffer and the protein and RNA complexes were eluted with TE buffer. The RNA was extracted using TRIzol reagent before real-time PCR analysis for SARS-CoV-2 or OC43 RNA.

### Immunofluorescence

Cells were fixed using 4% PFA for 30 min. After two PBS washes, cells were permeabilized with 0.2% Triton X-100/PBS before incubation with primary antibodies for 2 h at room temperature. Cells were washed in PBS, followed by incubation with secondary antibodies. Nuclei were stained with DAPI.

### In vitro transcription RNA assay.

Full RNA genome of SARS-CoV-2 was purified from supernatant of Vero E6 cells infected with SARS-CoV-2 by TRIzol (Thermo Fisher), 1 μg of RNA was reverse transcribed using the iScript cDNA synthesis kit (Bio-Rad). cDNA of the RNA genome of SARS-CoV-2 was used and amplified by PCR through primers with the T7 promoter sequence in the 5′ end for PCR to prepare templates of the in vitro transcription of the 5′ UTR, 3′ UTR and three other RNA fragments. The purified PCR products were used for genomic RNA fragment synthesis using a HiScribe T7 high yield RNA synthesis kit (NEB) according to the manufacturer’s instructions. The synthesized genomic RNA fragments were purified and labeled with biotin using the Label IT Biotin Labeling Kit (Mirus) for RNA pull-down assay and RIP assay with RNA competition. The sequences of primers with the T7 promoter sequence used in this study are listed in Table S1.

### Mice infection

All animal experiments were approved by the Institutional Animal Care and Use Committee at the University of Massachusetts Chan Medical School. Animals were kept in a specific pathogen-free (SPF) environment. The *Cnbp* KO and *Cnbp* Vavi-Cre conditional KO mice were generated as described previously^17^. *Ifnar* KO mice were obtained from Dr. Jonathan Sprent (Scripps). For SARS-CoV-2 infections, 12–16-week-old male and female mice were anesthetized with isoflurane and infected intranasally with 1 × 10^5^ PFUs of SARS-CoV-2 MA10 strain. Mice were monitored daily for weight loss and survival. Mouse organs were collected at indicated time points and placed in a bead homogenizer tube with 1 ml of DMEM + 2% FBS for homogenization, then 100 μl of this mixture was placed in PBS for tittering or in 300 μl Trizol LS (Invitrogen) for RNA extraction.

### Lung histology

Lungs were perfused with 10 U/mL heparin, then intratracheally inflated with 10% buffered-formalin and dissected from mice. Tissues were fixed in 4% paraformaldehyde overnight and embedded in 10% paraffin. Five micrometer thin sections were stained by H&E. Histomorphology, grading of histology scores, and evaluation of inflammation of each H&E slide were performed by Applied Pathology Systems.

### Flow cytometry

SARS-CoV-2 MA10 virus-infected mice were anesthetized at day 4 post infection. Mouse lung and spleen were collected and minced in RPMI and filtered through a 70 μm filter, then washed and resuspended in Red blood cell lysis buffer, then resuspendend in MACS buffer. Isolated lung and spleen mononuclear cells were stained with anti-CD64 BV711, anti-CD11b PE, anti-CD45.2, PerCP-Cy5.5, anti-Ly6G FITC, anti-MHCII PE-Cy7, anti-Ly6C APC, anti-Siglec-F AF700, and anti-F4/80 APC-Cy7. The stained cells were washed and resuspended in 4% PFA for 30 minutes. Cells were acquired on a Cytek Aurora cytometer. Flow cytometry analysis was done with the FlowJo software.

### Statistical analysis

GraphPad Prism 8 software (GraphPad Software, San Diego, CA) was used for data analysis using a two-tailed unpaired t-test. For mouse in vivo studies, 3 to 16 mice were used per experiment, Kaplan–Meier survival curves were generated and analyzed for statistical significance. A p-value of 0.05 was considered statistically significant (*p <0.05, **p<0.01, ***p < 0.001).

## Extended Data

## Supplementary Material

1
