# Supplementary material for: CNBP restricts SARS-CoV2 by regulating IFN and disrupting RNA-protein condensates
Source: Res Sq. 2022 May 2:rs.3.rs-1576788. Preprint. [Version 1] doi: 10.21203/rs.3.rs-1576788/v1 (PMC9094105; doi:10.21203/rs.3.rs-1576788/v1)
Supplement: 1 [file NIHPPRS1576788V1-supplement-1.pdf]

Gene	Forward primer	Reverse primer
**Q-PCR Primers**		
SARS-CoV2-N	CTCTTGTAGATCTGTTCTCTAAACGAAC	GGTCCACCAAACGTAATGCG
SARS-CoV2-Nsp14	TGGGGYTTTACRGGTAACCT	AACRCGCTTAACAAAGCACTC
HCoV-OC43-N	AGGAAGGTCTGCTCCTAATTC	TGCAAAGATGGGGAACTGTGGG
HCoV-OC43-M	GGCTTATGTGGCCCCTTACT	GGCAAATCTGCCCAAGAATA
RSV-A2	GCTCTTAGCAAAGTCAAGTTGAATGA	TGCTCCGTTGGATGGTGTATT
Human IFNb	GTCTCCTCCAAATTGCTCTC	ACAGGAGCTTCTGACACTGA
Human IFNa	CACACAGGCTTCCAGGCATTC	TCTTCAGCACAAAGGACTCATCTG
Human RSAD2	CTTTGTGCTGCCCCTTGAGGAA	CTCTCCCGGATCAGGCTTCCA
Human HPRT	ATCAGACTGAAGAGCTATTGTAATGA	TGGCTTATATCCAACACTTCGTG
murine IFNb	ATAAGCAGCTCCAGCTCCAA	CTGTCTGCTGGTGGAGTTCA
murine TNF-α	GGTGCCTATGTCTCAGCCTCTT	GCCATAGAACTGATGAGAGGGAG
murine IL12b	GGAAGCACGGCAGCAGAATA	AACTTGAGGGAGAAGTAGGAATGG
murine IL10	CGGGAAGACAATAACTGCACCC	CGGTTAGCAGTATGTTGTCCAGC
murine IL1b	CGGCACACCCACCCTG	AAACCGTTTTTCCATCTTCTTCT
murine GAPDH	TGGCAAAGTGGAGATTGTTGCC	AAGATGGTGATGGGCTTCCCG
**IVT Primers**		
5-UTR	TAATACGACTCACTATAGGGATTAAAGGTTTATACCTTCCCAG	AGA ACG TTC CGT GTA CCA AGC AA
3-UTR	TAATACGACTCACTATAGGGCAG TAG GGG AAC TTC TCC T	TTT TTG TCA TTC TCC TAA GAA GCT
5K	TAATACGACTCACTATAGGGCTCCACACGCAAGTTGT	ATT GGT TGC TCT GTG AAA TAA
10K	TAATACGACTCACTATAGGGTTCTGATGTTCTTTACCAA	ACC CTT GAT TGT TCT TTT CAC TGC
20K	TAATACGACTCACTATAGGGTTGATGGTCAAGTAGACTTA	ATC ACC AAT CAA AGT TGA ATC T
**sgRNAs**		
hCNBP sgRNA1	CACCGCCGTGTGCAGACCCGCGTG	AAACCACGCGGGTCTGCACACGGC
hCNBP sgRNA2	CACCGCGTCCGAGTCTCCGCCGCTG	AAACCAGCGGCGGAGACTCGGACGC
hCNBP sgRNA3	CACCGAAGACGGCTCGCAAGGTAG	AAACCTACCTTGCGAGCCGTCTTC
